# The Hospital Profession

**Published:** 1921-04-30

**Authors:** 


### The Hospital

The Workers' Newspaper of Administrative Medicine and Institutional Life, Administration, National Insurance and Health.

No. 1821, Vol. LXX. SATURDAY, APRIL 30, 1921. PRICE TWOPENCE

### THE HOSPITAL PROFESSION.

The desire to make hospital administration, in theory as well as in fact, a profession with a definite status and qualifications of its own has often been discussed in this country. It is now engaging the attention of America, to which allusion is made on page 76, where we summarise Dr. Goldwater's article in the March number of the *Modern Hospital.* It is easier to see the need for such a change than to decide upon the proper way to secure it. The first thing must be to determine the qualifications for the training of hospital administrators, and we await with interest the report on hospital training of the committee which the Rockefeller Foundation has appointed. There is a natural jealousy that the best posts in the hospital world should not generally be given to men of no previous administrative experience, and yet hospital committees do not always find such previous experience a decisive qualification. What, then, should the qualification include? Were the appointment of medical superintendents the rule the matter would be easy, since medical men are usually superintendents of certain institutions such as sanatoria and mental hospitals, and consequently a nucleus of qualified candidates already exists. Beside this, a medical qualification, while useful to the hospital administrator, also guarantees a certain standard of general education; and this perhaps influences those who claim that the chief administrative post in a hospital should always be held by a medical man. Indeed, it may be argued that, in theory at all events, the medical superintendent is the ideal man for the chief post. But nowadays the work of hospital superintendents is so complex that even where lay superintendents are employed a financial secretary is often appointed. Consequently, to favour the appointment of medical superintendents is not to relegate the layman to a back administrative seat.

From the layman, therefore, we should demand a certain educational standard, and make this depend upon some diploma, or preferably a degree, if the posts open to candidates are, as they ought to be, well enough paid to attract University graduates. This standard of general education determined, experience of accountancy and of institutional management remain. We presume that a gift for raising money is in the main a personal aptitude which cannot be taught. But nothing will be done to forward the advance of the profession until some uniformity is reached in the selection of candidates, for no one will much prize set educational qualifications if posts often go to those who have not bothered to attain them. Now that the British Hospitals Association has started its regional committees we think that one of the most useful subjects that they can discuss is the training and choice of hospital candidates.

